# Anti-cancer activity of ZnO chips by sustained zinc ion release

**DOI:** 10.1016/j.toxrep.2016.03.008

**Published:** 2016-03-19

**Authors:** Seong-Hee Moon, Won Jin Choi, Sik-Won Choi, Eun Hye Kim, Jiyeon Kim, Jeong-O Lee, Seong Hwan Kim

**Affiliations:** a Laboratory of Translational Therapeutics, Korea Research Institute of Chemical Technology, Daejeon 305-343, Republic of Korea; b Department of Strategy and Planning, Korea Institute of Science and Technology Information, Seoul 130-741, Republic of Korea; c Advanced Materials Division, Korea Research Institute of Chemical Technology, Daejeon 305-343, Republic of Korea; d Department of Biomedical Laboratory Science, School of Medicine, Eulji University, Daejeon 301-768, Republic of Korea

**Keywords:** ZnO, Zinc ion, Daunorubicin, Cancer

## Abstract

We report anti-cancer activity of ZnO thin-film-coated chips by sustained release of zinc ions. ZnO chips were fabricated by precisely tuning ZnO thickness using atomic layer deposition, and their potential to release zinc ions relative to the number of deposition cycles was evaluated. ZnO chips exhibited selective cytotoxicity in human B lymphocyte Raji cells while having no effect on human peripheral blood mononuclear cells. Of importance, the half-maximal inhibitory concentration of the ZnO chip on the viability of Raji cells was 121.5 cycles, which was comparable to 65.7 nM of daunorubicin, an anti-cancer drug for leukemia. Molecular analysis of cells treated with ZnO chips revealed that zinc ions released from the chips increased cellular levels of reactive oxygen species, including hydrogen peroxide, which led to the down-regulation of anti-apoptotic molecules (such as HIF-1α, survivin, cIAP-2, claspin, p-53, and XIAP) and caspase-dependent apoptosis. Because the anti-cancer activity of ZnO chips and the mode of action were comparable to those of daunorubicin, the development and optimization of ZnO chips that gradually release zinc ions might have clinical anti-cancer potential. A further understanding of the biological action of ZnO-related products is crucial for designing safe biomaterials with applications in disease treatment.

## 1 Introduction

Multifunctional zinc oxide (ZnO) has been used in various forms such as nanoparticles (NPs) or nanorods for biomedical applications including biosensing, imaging, drug delivery, and clinical implants [23], [31], [44], [46]. Since several studies have reported the toxicity of ZnO materials, their potential to induce cell death has been explored in cancer biology. The anti-cancer activity of ZnO nanomaterials has been described, and additive or synergistic effects of ZnO NPs with anti-cancer compounds (or drugs) on the induction of apoptosis in cancer cells also have been reported [10], [43].

The effects of zinc ions might explain ZnO-induced cytotoxic and apoptotic activity. Zinc ions released from ZnO materials induce oxidative stress-mediated cell death [3], [6], [30], [40], and the strong correlation between ZnO NP-induced cytotoxicity and free zinc ion concentration also suggests a requirement for ZnO dissolution for effective cytotoxicity [36]. Consistently, extracts exhibit more cytotoxicity in suspended cells than do nanostructured ZnO chip coatings *per se* [30]. These findings motivated us to fabricate a ZnO chip that gradually releases zinc ions and to evaluate its anti-cancer activity. The cytotoxicity of the ZnO chip was compared to that of daunorubicin (an anti-cancer drug used to treat leukemia) in human B lymphocyte Raji cells. After using inductively coupled plasma (ICP) atomic emission spectroscopy (AES) to analyze the zinc ion concentration released from the ZnO chip, we evaluated the cytotoxicity of zinc ions released from the chip in Raji cells and human peripheral blood mononuclear cells (PBMCs). In addition, using a cellular peroxidase activity assay, antibody array, and Western blot analysis, we investigated the mechanism underlying the anti-cancer activity of zinc ions.

## 2 Materials and methods

### 2.1 Fabrication of the ZnO chip

ZnO chips were fabricated by coating ZnO onto circular glass slides that fit in a 6-well culture plate using an atomic layer deposition (ALD) method. ALD is a thin film-coating method based on the sequential use of a gas-phase chemical reaction. A single cycle of ALD consists of a pulse of diethyl zinc (DEZ) followed by a purge process. This step is then followed by a subsequent pulse of water (oxidant) to form a layer of ZnO. The ALD method enables atomic-scale control of the thickness of ZnO thin films, with a chip undergoing 200 cycles of this process characterized as a 200-cycle ZnO chip. ALD was performed in a Lucida D-100 chamber using DEZ (Sigma-Aldrich, MO, USA) with water as the reactant and oxidant, respectively. ALD was carried out under full saturation conditions, with DEZ–purge–water–purge cycles controlled at 0.5 s–10 s–0.1 s–30 s. The deposition temperature was fixed at 150 °C.

### 2.2 ICP-AES analysis

After the ZnO chip was incubated with cell culture media, the concentration of zinc ions in the media was analyzed using an iCAP 6500 (Thermo Scientific, MA, USA).

### 2.3 Cell culture

A549 cells and Raji cells were purchased from ATCC (VA, USA) and cultured with DMEM and RPMI-1640 containing 10% heat-inactivated fetal bovine serum and 1% antibiotics (100 U/ml penicillin and 100 μg/ml streptomycin) in a humidified atmosphere of 5% CO_2_ at 37 °C, respectively. All cell culture materials were purchased from Hyclone (UT, USA). Human PBMCs and culture medium were purchased from Zen-Bio Inc. (NC, USA).

### 2.4 Apoptosis analysis

Cells (2 × 10^5^ cells/ml) were incubated in the culture plate with the ZnO chip or with daunorubicin (Selleckchem, TX, USA) or the extracts containing zinc ions, for the indicated time. Then, using Muse™ Annexin V and the Dead Cell Assay kit (Millipore, MA, USA), live and apoptotic populations were measured in a Muse™ Cell Analyzer (Millipore).

### 2.5 Caspase assay

Caspase activity was measured using the Muse™ MultiCaspase kit (Millipore) according to the manufacturer’s protocol. This pan-caspase assay can detect the presence of multiple caspases (*i.e.*, caspase-1, 3, 4, 5, 6, 7, 8, and 9). Briefly, Raji cells (2 × 10^5^ cells/ml) were incubated in the culture plate with the ZnO chip or with daunorubicin for 24 h and harvested. Then, live caspase-positive and necrotic populations were measured in the Muse™ Cell Analyzer.

### 2.6 Propidium iodide (PI) staining

Raji cells (2 × 10^5^ cells/ml) were incubated with a 400-cycle ZnO chip or with daunorubicin (100 μM) for 24 h. Then, cells were stained with 1 μg/ml PI (Clontech, CA, USA) in the dark at room temperature for 15 min, and images of cells were captured at a magnification of ×40 in an ImageStream flow cytometer (Amnis, WA, USA).

### 2.7 Conductivity and surface roughness measurement

Hall measurements were conducted using the HMS-3000 (Ecopia, South Korea) in the Van der Pauw configuration. To ensure proper electrical measurement, the induced current was fixed to 1 mA. A thermal annealing treatment was conducted on a hot plate. A Dimension 3100 atomic force microscope (AFM; Veeco, NY, USA) was employed to obtain topological images and confirm surface roughness.

### 2.8 Cellular peroxidase activity assay

Cellular peroxidase activity was measured using the Amplex Red Hydrogen Peroxidase assay kit (Invitrogen, CA, USA) according to the manufacturer’s protocol. Briefly, Raji cells (2 × 10^5^ cells/ml) were incubated with or without a 400-cycle ZnO chip for the indicated time, and the cellular peroxidase activity then was measured at _Ex_530 nm and _Em_590 nm in a Wallac EnVision microplate reader (PerkinElmer, MA, USA).

### 2.9 Oxidative stress measurement

Oxidative stress was measured using the Muse™ Oxidative Stress kit (Millipore) according to the manufacturer’s protocol. Briefly, Raji cells (2 × 10^5^ cells/ml) were incubated in the culture plate with a ZnO chip or with daunorubicin for the indicated time. Then, live cells and cells with signs of ROS were distinguished and counted in the Muse™ Cell Analyzer.

### 2.10 Western blotting analysis

In the absence or presence of zinc ions (10 mg/l), Raji cells (5 × 10^5^ cells/ml) were incubated for 3 or 12 h, washed with ice-cold phosphate-buffered saline, and lysed in lysis RIPA buffer (Elpis Biotech, Daejeon, Korea) containing 1× protease and phosphatase inhibitors (Pierce Biotechnology, IL, USA). Cell lysates were subsequently incubated at 4 °C for 20 min and then centrifuged at 14,000*g* for 15 min. The protein concentration of the supernatants was determined using a protein assay kit (Bio-Rad, CA, USA), and denatured proteins (20 μg) were loaded in a 10% SDS–PAGE gel, followed by transfer by electroblotting to a PVDF membrane (Millipore, CA, USA). Membranes were incubated with the blocking buffer consisting of TBST buffer (10 mM Tris–HCl pH 7.5, 150 mM NaCl, 0.1% Tween 20) and 5% nonfat dry milk for 2 h, and then incubated with the primary antibodies diluted with the blocking buffer (1:1000) overnight at 4 °C. The membranes were washed with TBST at room temperature and incubated with diluted secondary antibodies (1:5000) for 1 h. After being washed with TBST three times (15 min each), the membranes were developed with the SuperSignal West Femto Maximum Sensitivity Substrate (Pierce Biotechnology) using a LAS-3000 luminescent image analyzer (Fuji Photo Film Co., Ltd., Japan). The antibody against actin and secondary antibodies were purchased from Santa Cruz Biotechnology (CA, USA). All other antibodies were purchased from Cell Signaling Technology (MA, USA).

### 2.11 Human apoptosis array analysis

Human apoptosis array analysis was carried out according to the manufacturer’s protocol. Briefly, in the absence or presence of zinc ions (10 mg/l), Raji cells (5 × 10^5^ cells/ml) were incubated for 3 or 12 h and solubilized by lysis buffer. After protein quantification, cell lysates (400 μg) were adjusted to 1.5 ml with lysis buffer and probed to the pre-coated Human Apoptosis Array membrane (Human Apoptosis Array, R&D Systems, MN, USA) at 4 °C overnight. Then, array dots in the membrane were developed and quantified with the LAS-3000 luminescent image analyzer (Fuji Photo Film Co., Ltd., Japan). Densitometric analysis was performed using ImageJ software and the relative, normalized expression level of each molecule to the reference spots presented.

### 2.12 Statistical analysis

All quantitative values are presented as mean ± SD. Statistical differences were analyzed using Student’s *t* test. A value of *P* < 0.05 was considered significant.

## 3 Results and discussion

### 3.1 Fabrication and characterization of ZnO chip

**Fig. 1:**
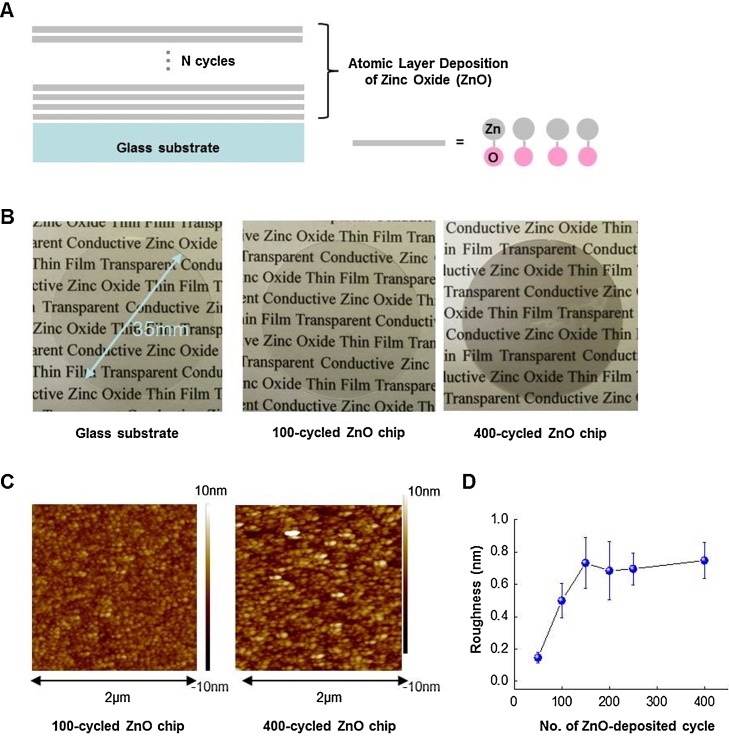
Growth of ZnO thin films and its characteristics. (A) A schematic depiction of ALD-processed ZnO thin film with different thickness. (B) A 6-well plate-sized glass substrate was used to prepare chips deposited by ZnO thin films. The transparent 100-cycle and 400-cycle ZnO chips are shown. Also shown are topological images (C) and a plot of surface roughness (D), analyzed by AFM, *versus* the number of ALD cycles, respectively.

ZnO thin films were deposited on glass substrates using the ALD method (Fig. 1A). As shown in Fig. 1B, ZnO chips are optically transparent. To investigate the surface characteristics of ZnO chips, we used AFM in non-contact mode. AFM images of ZnO chips deposited in different numbers of ALD cycles are shown in Fig. 1C, and the analysis of the surface roughness is shown in Fig. 1D.

### 3.2 Potential of ZnO chip to release zinc ions and induce apoptosis in Raji cells

**Fig. 2:**
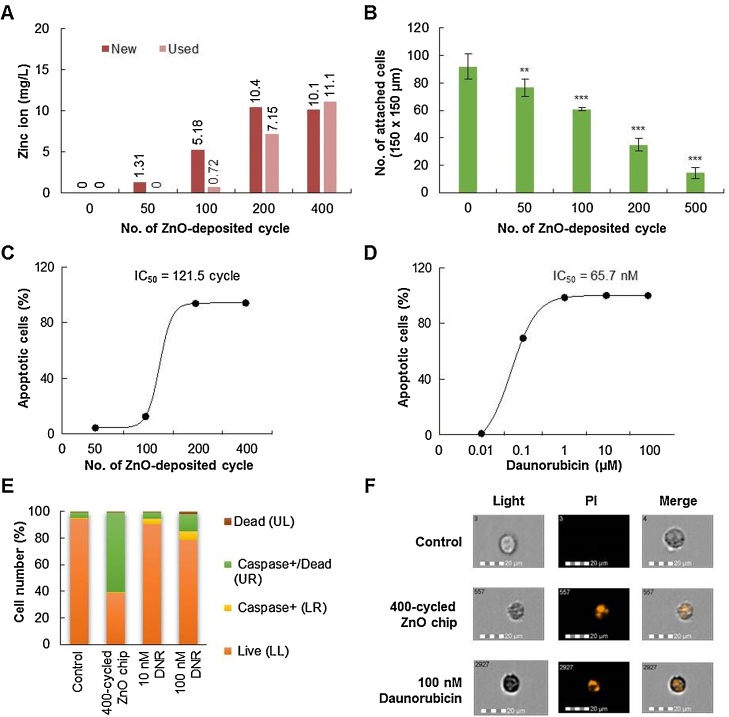
ZnO chips release zinc ions and induce apoptosis of Raji cells in a ZnO deposition cycle-dependent manner. (A) Concentration of zinc ions in media incubating chips for 24 h was measured by ICP analysis (presented as ‘new’). The used chips were re-incubated with the fresh media for an additional 24 h, and the concentration of zinc ions in media was measured (presented as ‘used’). (B) Monolayer A549 cells were seeded onto ZnO chips. After 24 h of incubation, non-adherent cells were discarded, and attached cells were counted under a light microscope. **, *P* < 0.01; ***, *P* < 0.001 *versus* chips without ZnO. (C) The apoptotic activity of ZnO chips in Raji cells was evaluated. Raji cells were incubated on built-in various cycles of ZnO chips for 24 h, and then the number of apoptotic cells was measured in a Muse™ Cell Analyzer by using Muse™ Annexin V and the Dead Cell Assay kit. The IC_50_ value was determined from the ZnO deposition cycle-dependent apoptotic cell number curve using GraphPad Prism software (GraphPad Software, Inc., CA, USA). (D) The apoptotic activity of daunorubicin was also evaluated in Raji cells. The IC_50_ value was determined from the concentration-dependent apoptotic cell number curve using GraphPad Prism software. (E) The effect of 400-cycle ZnO chips or daunorubicin (DNR) on the induction of caspases was measured in Raji cells by using the Muse™ MultiCaspase kit. The percent of caspase-positive cells is given. (F) Representative images of nuclear changes in Raji cells after incubating with 400-cycle ZnO chips or 100 μM daunorubicin. Nuclear fragmentation was observed by PI staining.

The amount of zinc ions released from the ZnO chip in the culture media was evaluated by ICP-AES (Fig. 2A). When ZnO chips were incubated in culture media (5 ml) in a 6-well culture plate for 24 h, the amount of released zinc ions gradually increased according to the ZnO deposition cycle number, but after 200 cycles, it was likely to be saturated to 10 mg/l. When the used ZnO chips (that were incubated with the culture media for 48 h) were re-incubated for 24 h after being washed in phosphate-buffered saline, zinc ions were still released from films, and consistent with new chips, saturation was likely to 10 mg/l. These results suggested that ZnO thin films on the chip could continuously and slowly release zinc ions and that the amount released might be isotonically saturated.

Next, we investigated whether the ZnO chip could affect the viability of cancer cells. First, a monolayer of human lung epithelial adenocarcinoma A549 cells was applied to ZnO chips. When A549 cells were seeded onto ZnO chips in a 6-well culture plate, the number of attached A549 cells was significantly decreased according to the number of ZnO deposition cycles (Fig. 2B). Considering that there was no difference in the surface roughness between 150–400 deposition cycles (Fig. 1D), this result suggested that the physicochemical properties of ZnO chips might affect the attachment of A549 cells, including the sustained release of zinc ions.

Because cell type-dependency or other complicated physicochemical properties of ZnO in aqueous conditions could affect the attachment of A549 cells, the suspended Raji cells were chosen and applied for evaluation of the anti-cancer activity of ZnO chips. Raji cells were incubated in the culture plate with ZnO chips or with daunorubicin for 24 h, then stained with Annexin V and the Dead Cell kit, and acquired on the cell analyzer. As shown in the plots (Supplementary Fig. 1A and B), viable cells (lower left), cells in the early stages of apoptosis (lower right), cells in the late stages of apoptosis or dead by apoptotic mechanisms (upper right), and cells that had died *via* necrosis but not through the apoptotic pathway (upper left) were displayed as dots. Glass slides did not affect the viability of suspended Raji cells, but ZnO deposition increased the number of apoptotic cells in a ZnO deposition cycle-dependent manner. Although ZnO-mediated toxicity to cancer cells has been suggested to result from direct contact between materials and cells [26], the apoptosis of the suspended Raji cells in the ZnO chip might be explained by the effect of zinc ions released from the chip. The half-maximal inhibitory concentration (IC_50_) of the ZnO chip on the viability of Raji cells was 121.5 cycles (Fig. 2C), and that of daunorubicin was 65.7 nM (Fig. 2D). Thus, a 121.5-cycle ZnO chip could clinically exhibit anti-cancer activity similar to that of 65.7 nM daunorubicin. Furthermore, the number of apoptotic Raji cells was dramatically increased to 98% in the culture condition with a 200-cycle ZnO chip or 1 μM daunorubicin.

In addition, as shown in Supplementary Fig. 1C, the MultiCaspase assay revealed that the apoptotic activity of the ZnO chip was strongly dependent on the induction of caspases; when Raji cells were cultured with a 400-cycle ZnO chip, 60% of cells were caspase-positive (Fig. 2E). Also, Raji cells incubated with the ZnO chip and daunorubicin displayed nuclear fragmentation, a hallmark of apoptosis (Fig. 2F).

### 3.3 Apoptotic activity of the ZnO chip because of released zinc ions, not electrical conductivity

**Fig. 3:**
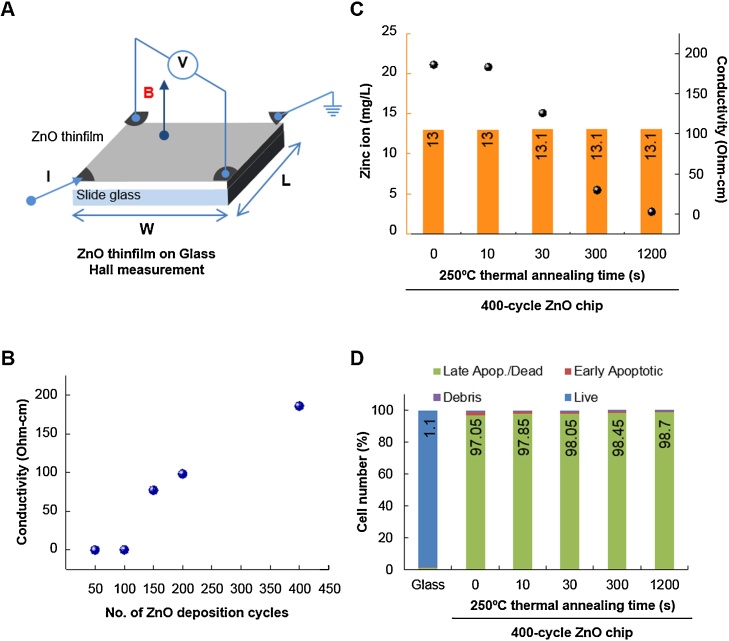
Apoptotic activity of ZnO chips is due to the released zinc ions, not electrical conductivity. (A) A scheme of Hall’s measurement. (B) The electrical conductivity of ZnO chips with respect to the number of ALD cycles. (C) The conductivity of 400-cycle ZnO chips was measured after 250 °C thermal annealing treatment (black dots). The amount of released zinc ions in 400-cycle ZnO chips after thermal annealing treatment was measured by ICP analysis after incubation with culture media for 24 h (orange bars). (D) The effect of thermal annealing time on the number of apoptotic Raji cells was evaluated using the Muse™ Cell Analyzer. Cells were incubated with chips for 24 h. The percent of apoptotic cell number is presented.

Having shown that zinc ions were sustainably released in a ZnO deposition cycle-dependent manner and demonstrated apoptosis of Raji cells, we thought that it was highly likely that the released zinc ions were responsible for the observed Raji cell apoptosis. However, another possibility was electrical conductivity of the ZnO chips. In ALD-grown ZnO thin films, electrical conductivity of the film increases in a ZnO deposition cycle-dependent manner as well. Although the electrical conductivity of ZnO chips is unlikely to have affected the viability of suspended Raji cells, we nevertheless addressed the possibility. As shown in Fig. 3A, when the electrical conductivity of ZnO chips was measured, it was increased in a ZnO-deposited cycle-dependent manner (Fig. 3B). Electrical conductivity of ZnO can be tuned by post-annealing [29], and it is possible to fabricate ZnO thin films with the same thickness but dramatically different electrical conductivities. When 400-cycle ZnO thin film was annealed at 250 °C, its conductivity was gradually decreased by increasing thermal annealing time (black dots in Fig. 3C). However, the change in thermal annealing time in a 400-cycle ZnO chip did not change the amount of released zinc ions (orange bars in Fig. 3C) or the number of apoptotic Raji cells (Fig. 3D). In the 400-cycle ZnO chip, 13 mg/l of zinc ions was released and induced apoptosis in 98% of Raji cells. These results confirm that the apoptotic activity of the ZnO chip did not originate from electrical conductivity.

### 3.4 Selective apoptotic activity of ZnO chips

**Fig. 4:**
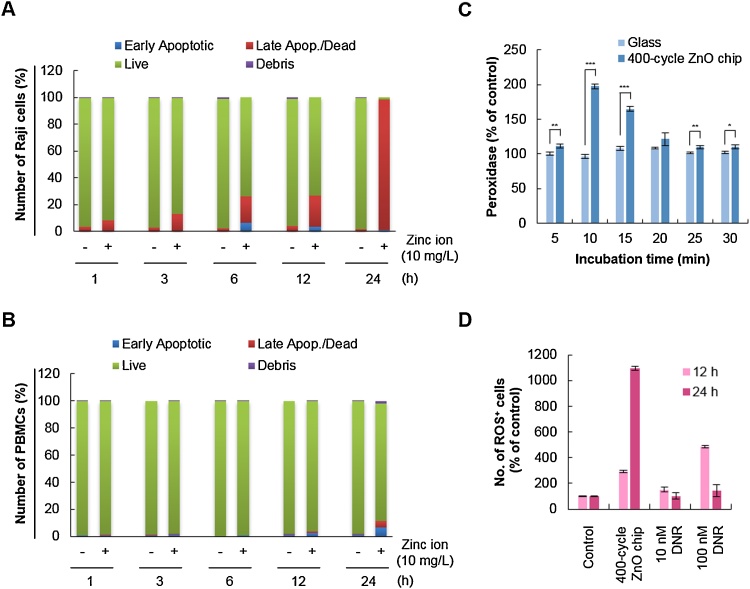
ZnO chips selectively induce apoptosis of Raji cells with induction of ROS. Apoptotic activity of zinc ions released from chips was evaluated in Raji cells (A) and PBMCs (B) using the Muse™ Cell Analyzer. (C) ROS generation was evaluated with the Amplex Red Hydrogen Peroxidase assay kit after Raji cells were cultured with glass substrate or a 400-cycle ZnO chip. *, *P* < 0.05; **, *P* < 0.01; ***, *P* < 0.001. (D) Oxidative stress was measured using the Muse™ Oxidative Stress kit. The percent of cells exhibiting ROS in the red M2 gate (Supplementary Fig. 2) is presented.

Another question in the context of the anti-cancer activity of the ZnO chip is whether it affects the viability of normal cells. Therefore, the effect of the ZnO chip on the viability of Raji cells was compared to that of normal PBMCs. Because of the dramatic induction of apoptosis in Raji cells with the 200-cycle ZnO chip and the release of 10 mg/l zinc ions (Fig. 2A), we incubated Raji cells and PBMCs with culture media containing 10 mg/l zinc ions for 1–24 h and analyzed cell apoptosis. As shown in Fig. 4A and B, 10 mg/l zinc ions time-dependently increased the number of apoptotic Raji cells, but not PBMCs, suggesting that the ZnO chip could selectively induce apoptosis in cancer cells rather than in normal cells. Indeed, ZnO NPs consistently have been reported to selectively (or preferentially) induce apoptosis in cancer cells rather than normal cells [3], [19]. The selectivity of ZnO-related biomaterials should be considered before they are applied for a clinical purpose.

### 3.5 ROS-inducing activity of ZnO chips in Raji cells

A remaining question was how zinc ions trigger apoptosis in Raji cells. To elucidate the mechanism underlying the anti-cancer activity of the ZnO chip, we carried out the cellular peroxidase activity assay to indirectly measure the level of hydrogen peroxide generated by zinc ions. Oxidative stress resulting from reactive oxygen species (ROS) induces cancer cell apoptosis [22], [28] and ZnO NPs increase the production of hydrogen peroxide, a ROS, to induce cell death [9], [17]. Here, we found that cellular peroxidase activity was significantly induced when Raji cells were incubated with a 400-cycle ZnO chip (Fig. 4C). As shown in Supplementary Fig. 2, ROS-negative live cells in the blue M1 gate and ROS-positive cells in the red M2 gate were counted using the Oxidative Stress assay based on the intracellular detection of superoxide radicals. When Raji cells were incubated with the 400-cycle ZnO chip for 24 h, 67% of cells were ROS-positive while about 47% of cells treated with 100 nM daunorubicin for 12 h were ROS-positive (Fig. 4D). These findings suggested that the increase in cellular oxidative stress by the ZnO chip could be involved in its apoptotic activity

### 3.6 Zinc ions down-regulate anti-apoptotic molecules in Raji cells

**Fig. 5:**
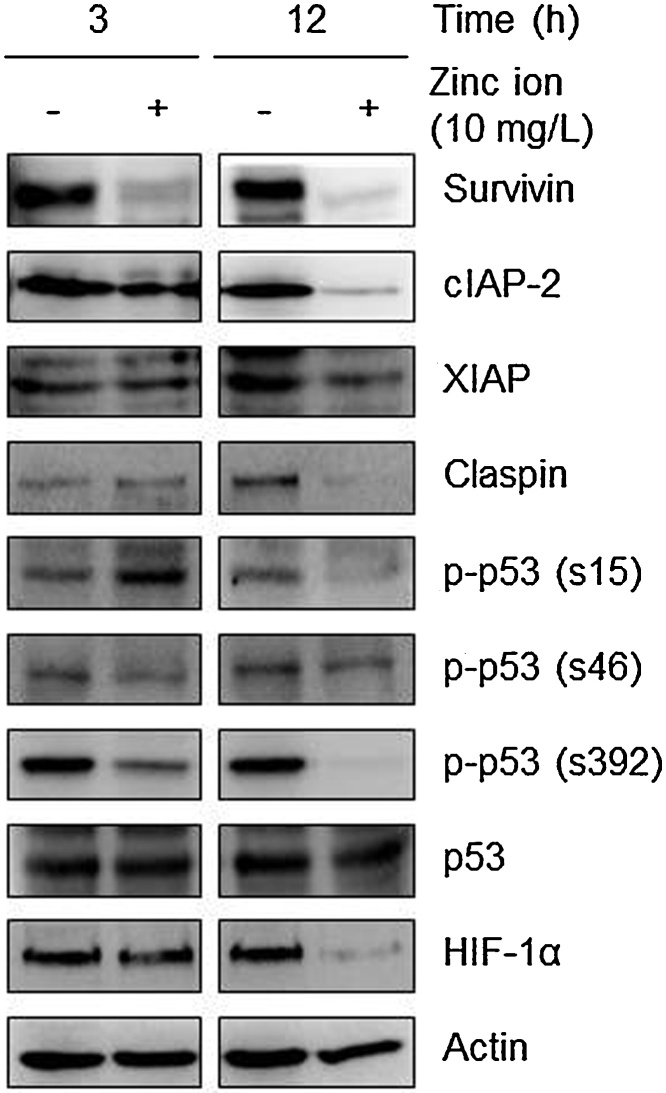
Zinc ions down-regulate anti-apoptotic proteins. The effects of zinc ions on the expression of apoptosis-related proteins were confirmed by Western blot analysis. Actin was used as a loading control.

Next, the nitrocellulose membrane array containing 35 different capture antibodies printed in duplicate was applied for evaluation of the effect of zinc ions on the expression levels of apoptosis-related proteins. As shown in Supplementary Fig. 3, the antibody-based apoptosis array analysis revealed that zinc ions down-regulated protein levels of anti-apoptotic molecules, including inhibitor of apoptosis proteins (IAPs), claspin, and phosphorylated forms of p53 and hypoxia inducible factor (HIF)-1α in Raji cells. The down-regulation of anti-apoptotic molecules by zinc ions were further confirmed by Western blot analysis (Fig. 5).

Consistent with the report showing the significant down-regulation of survivin by ZnO nanorods in cancer cells [2], the protein level of survivin in Raji cells was dramatically decreased by incubation with zinc ions for 3 h. This rapid reduction could be the result of proteasomal degradation because the proteasomal degradation of survivin has been reported as an apoptotic mechanism in cancer cells [11], [15], [37].

Zinc ions also down-regulated the protein level of cIAP-2 but not of X chromosome-linked IAP (XIAP) in Raji cells. Selective IAP down-regulation by pharmacological inhibition has been recently suggested to enhance the sensitization of leukemia cells to tumor necrosis factor-related apoptosis-inducing ligand (TRAIL)-induced apoptosis [47]. Because anti-cancer compounds can potentiate TRAIL-mediated death of cancer cells accompanied by induction of ROS and the down-regulation of anti-apoptotic molecules including survivin, cIAPs, and XIAP [20], ROS-generating ZnO materials might be used as a potent TRAIL sensitizer for cancer treatment [32], [45].

In addition, claspin might be used to determine the sensitivity of cancer cells to zinc ions [21]. Cleavage and degradation of claspin during apoptosis have been reported [34], and recently, it has been shown to positively affect the survival of cancer cells [7].

Oxidative stress and cytotoxicity induced by ZnO nanomaterials also could be explained by the status of p53. Mutant p53 proteins that impair tumor suppressor function are seen in 50% of human cancers [18], commonly called ‘mutant p53 gain of oncogenic function’. One of the potential molecular mechanisms underlying the gain of function of mutant p53 is a role as an oncogenic transcription factor [38]. Apparently, mutant p53 exhibits increased tumorigenicity in vivo and enhances resistance to anti-cancer drugs used clinically [1], [5]. Moreover, post-translational modification of mutant p53, such as phosphorylation, has been suggested to contribute to tumorigenesis [39]. Of importance, the phosphorylation of mutant p53 at Ser392 regulates its oncogenic function [42]; moreover, inhibition of mutant p53 phosphorylation at Ser15 or Ser315 has been suggested to restore its tumor suppressor function [39]. As is typical for Burkitt lymphoma, Raji cells used in this study have a p53 mutation [12]. Therefore, the zinc ion-mediated down-regulation of p-p53 in Raji cells could lead to apoptosis *via* reduced oncogenic activities. Similar to our results, ZnO NPs induce oxidative stress-mediated apoptosis in cancer cells *via* the p53 pathway [3]. In addition, p53 deficiency enhances susceptibility to ZnO-induced cancer cell death in a ROS-dependent manner [35], but here, the total p53 expression level was not changed by zinc ions.

The transcription factor HIF-1α regulates the expression of numerous genes involved in cellular metabolism, cell cycle regulation, angiogenesis, and inhibition of apoptosis. HIF-1α and its signaling pathway in cancer cells are related to the role of p53 and/or ROS [16], [33]. In addition, HIF-1α is activated aberrantly in hematological malignancies [8], [14], and inhibition of its activity correlates with sensitization of chemo-resistant cells to apoptosis [27]. Here, incubation with zinc ions for 12 h strongly down-regulated the protein expression level of HIF-1α. Inhibiting HIF-1α signaling by reducing its protein level or its transcriptional activity has been considered as an anti-cancer strategy to overcome drug resistance to chemotherapy [41].

## 4 Conclusion

**Fig. 6:**
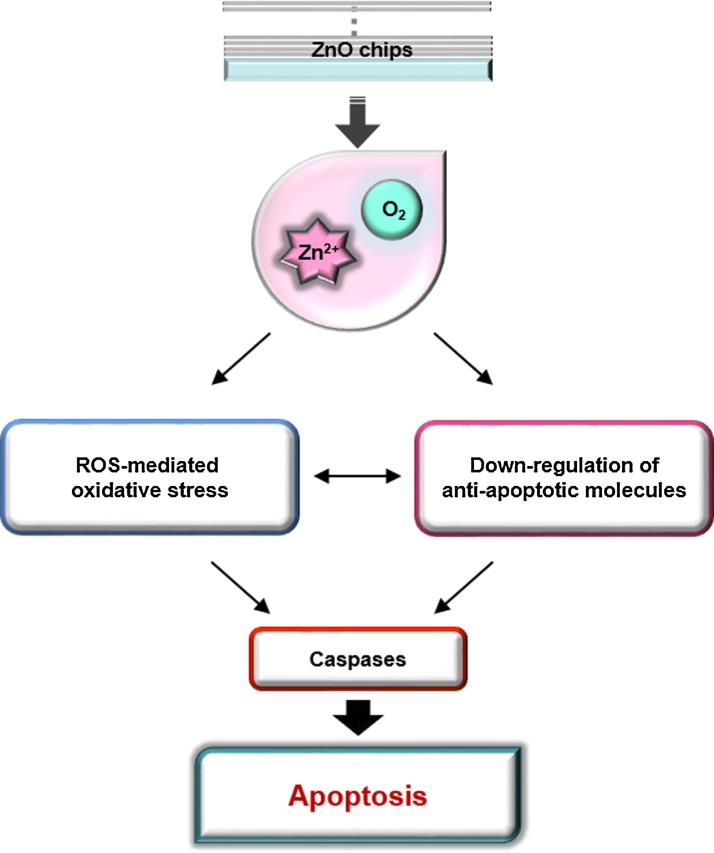
A schematic model presenting the ZnO chip’s biological potential to induce apoptosis of cancer cells.

In summary, we fabricated chips deposited with ZnO thin films and characterized their anti-cancer activity by the release of zinc ions in a ZnO deposition cycle-dependent manner, independent of conductivity. The caspase-dependent apoptotic activity of ZnO chips could be the result of the potential of zinc ions to induce oxidative stress and down-regulate protein levels of anti-apoptotic molecules including IAPs, claspin, p-p53, and HIF-1α in Raji cells (Fig. 6). Through apoptosis analysis, the IC_50_ of ZnO was for the first time quantitatively assessed as 121.5 cycles. Because oral administration of ZnO NPs can cause injury to the liver, kidney, and lung in healthy adult mice [13], a ZnO chip with a gradual release of zinc ions might be applied topically to inhibit skin cancers [4]. In addition, along with cream-based topical therapeutics, a patch-based version of the ZnO chip including anti-cancer drugs might additively or synergistically induce apoptosis of skin cancers [24], [25]. Furthermore, ZnO *per se* might be used as a coating material for orthopedic and dental implants to inhibit bacterial adhesion and promote osteoblast growth [23]. An understanding of the cytotoxicity of ZnO-related products is crucial for the further design of safe biomaterials for their application in treating diseases like cancer.

## Transparency document

Transparency Document
