# Supplementary material for: Anti-cancer activity of ZnO chips by sustained zinc ion release
Source: Toxicol Rep. 2016 Mar 19;3:430–8. doi: 10.1016/j.toxrep.2016.03.008 (PMC5615914; doi:10.1016/j.toxrep.2016.03.008)
Supplement: Supplementary file 2 [file mmc1.docx]

Supporting Information

**Anti-Cancer Activity of ZnO Chips by Sustained Zinc Ion Release**

*S. -H. Moon^a,b,†^, W. J. Choi^c,†^, S. –W. Choi^a^, J. Kim^d^, E. H. Kim^a^, J. -O. Lee^c,*^ and S. H. Kim^a,*^*


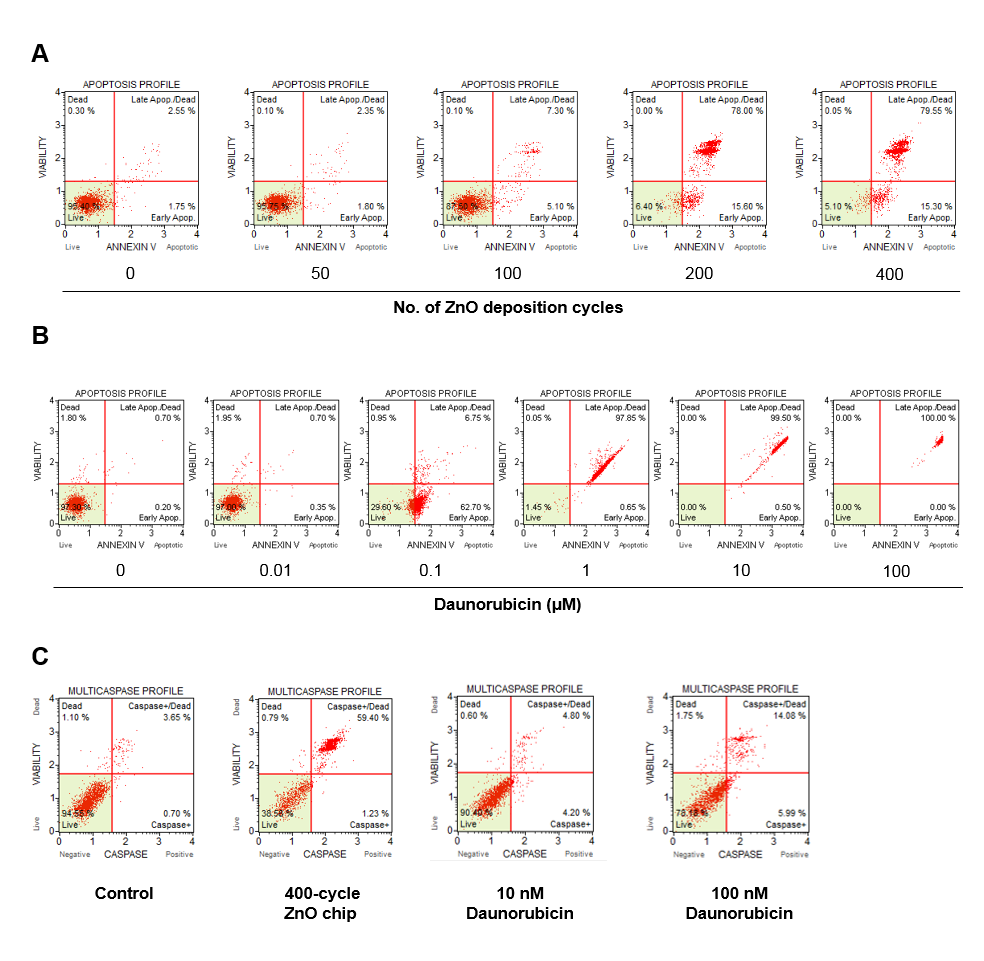


**Supplementary Figure 1.** The apoptotic activities of ZnO chips (A) and daunorubicin (B) in Raji cells were evaluated. Raji cells were incubated with ZnO chips or daunorubicin for 24 h, and then the number of apoptotic cells was measured with a Muse^TM^ Cell Analyzer by using Muse^TM^ Annexin V and the Dead Cell Assay Kit. (C) The effect of 400-cycle ZnO chips or daunorubicin on the induction of caspases was measured in Raji cells by using the Muse^TM^ MultiCaspase kit.


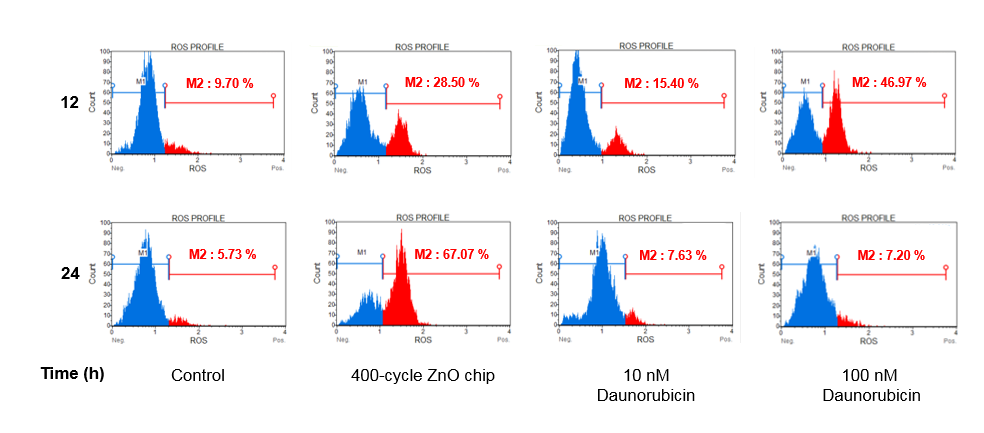


**Supplementary Figure 2.** The effect of 400-cycle ZnO chips or daunorubicin on ROS induction in Raji cells was measured with the Muse^TM^ Oxidative Stress kit. The percent of cells exhibiting ROS in the red M2 gate is presented.


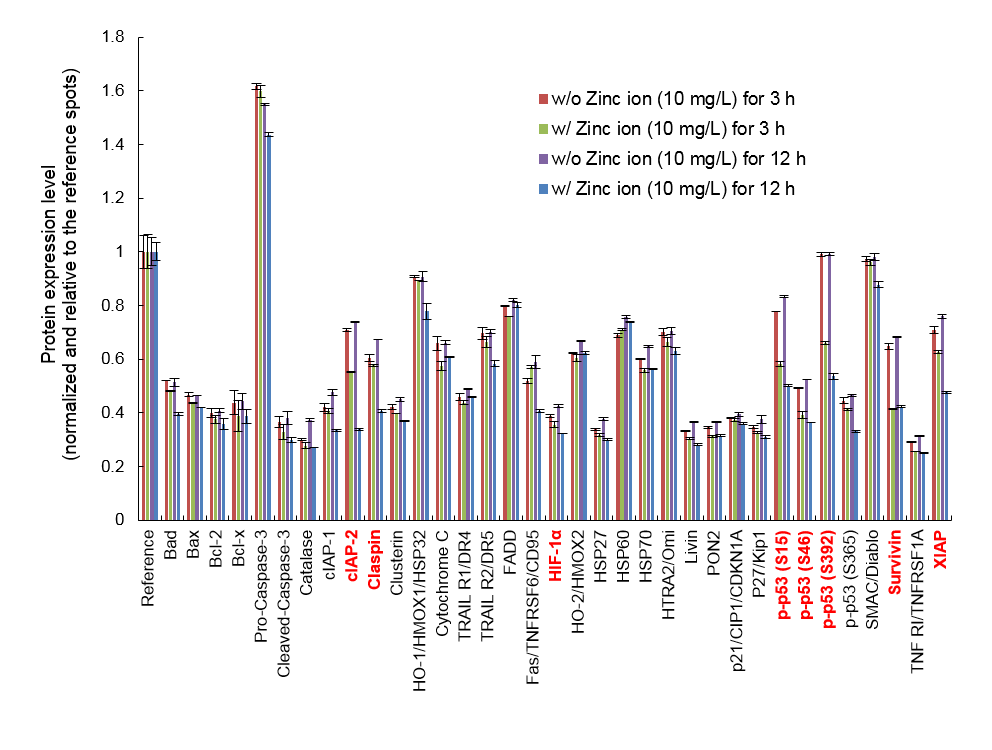


**Supplementary Figure 3.** The effects of zinc ions on the expression of apoptosis-related proteins were evaluated using the Human Apoptosis Array. Spot pixel levels were analyzed densitometrically with a LAS-3000 luminescent image analyzer and then normalized by the reference spots.
